# A p53 peptide mucosal vaccine induces cellular and humoral immunity and anti-tumor effects in a murine colorectal cancer model

**DOI:** 10.1038/s41417-026-01035-6

**Published:** 2026-04-25

**Authors:** Su He Wang, Zhengyi Cao, Katarzyna W. Janczak, Ying Feng, Maranne Green, Shengzhuang Tang, Eric R. Fearon, James R. Baker

**Affiliations:** 1 Department of Internal Medicine, University of Michigan Medical School, Rogel Cancer Center, Ann Arbor, MI USA; 2 Rogel Cancer Center, Human Genetics, University of Michigan Medical School, Ann Arbor, MI USA; 3 Rogel Cancer Center, Pathology, University of Michigan Medical School, Ann Arbor, MI USA

**Keywords:** Cancer, Drug development

## Abstract

Many patients with metastatic and recurrent colorectal cancer (CRC) have poor outcomes, due to resistance to current treatment approaches. Missense mutations in the *TP53* gene are found in about 65% of CRCs, but no current treatment approach targets mutant *TP53* activity. To develop a vaccine approach against CRCs expressing a mutant p53 protein, we used a murine CRC model with conditional expression of a *Trp53* codon R270H missense allele, and immunized mice with the experimental vaccine, which combined a synthetic R270H p53 peptide and wild-type p53 recombinant protein with a mucosal nanoemulsion (NE) adjuvant. The p53/NE vaccine was administered intranasally to the mice after mutant p53 induction and tumor initiation. Vaccinated mice had markedly increased serum anti-p53 IgG, IgG2a, and IgG2b as compared to control mice. The vaccination also enhanced antigen-specific Th1 and Th17 cellular immune responses, as shown by increasing production of IFNγ, IL-17a and IL-2. The immunized animals had significantly decreased tumor size, prolonged survival and increased tumor CD8^+^ T cell infiltrates. Collectively, we have demonstrated a mucosal vaccine against mutated p53 protein in CRC can induce antigen-specific cellular and humoral immunity. The findings suggest potential value in pursuing mutated p53 as a vaccine target in CRC.

## Introduction

Colorectal cancer (CRC) is among the most common cancer types worldwide. The overall 5-year survival rate for CRC patients is around 60%, despite progress in advancing multi-disciplinary treatment approaches and new drug regimens [1, 2]. Recently, immunotherapy has emerged as an option for treatment in a subset of CRC patients. However, immunotherapy with immune checkpoint inhibitors (ICI) is effective in the 15% of CRC patients who have high-frequency microsatellite instability (MSI-H). The absence of response to ICI also appears associated with an immune-suppressive tumor microenvironment (TME) in CRCs. [3, 4]. The poor response to ICIs in most CRC patients has led to interest in novel approaches to enhance anti-CRC immunity.

Cancer vaccines are one method to enhance immunity against solid tumors and have been shown to induce immune responses against cancers in experimental settings. Peptide vaccines provide a potential approach to generate tumor-specific immune responses [2]. Despite this, several studies with anti-cancer peptide vaccines have not shown benefit in CRC treatment. Lynch and colleagues reported vaccination with carcinoembryonic antigen (CEA) and HER2/neu (HER2) mutant peptides induced immune responses in 70% of patients with advanced CRC but failed to demonstrate clinical benefits [5]. Kim and colleagues used a telomerase peptide vaccine (GV1001) in patients with metastatic colorectal cancer, but no specific GV100 immune response was observed [6]. Schoen and colleagues conducted a multicenter, double-blind, placebo-controlled trial with a mucin 1 (MUC1) peptide vaccine in patients with advanced CRC, and although an immune response was observed in vaccine recipients, tumor recurrence was not suppressed [7]. Despite these disappointing results, peptide vaccine immunotherapy in model CRC systems continue to suggest the potential for benefit. Recently, Shi and colleagues evaluated a cancer-testis antigen ODF2-drived peptide vaccine in MSS-CRC tumor-bearing NOD/SCID mice and found the induction of peptide-specific CD8^+^ T cells in vitro and in vivo [8]. As such, cancer vaccine approaches in human CRC may require either different target neoantigens or immunological approaches.

When examining the failure of immunotherapy in CRC antigen selection is an issue. Most tumor antigens have limited expression or other problems. HER2 overexpression is present in only 3–5% of cases of CRC [9]. CEA is released from cancer cells and is also present at elevated levels in nonmalignant medical conditions [10]. Both MUC1 and ODF2 are also strongly expressed in normal tissues providing tolerogenic signals [11, 12]. Also, there are problems with current immune approaches to CRC. None of the current CRC vaccines target mucosal sites, and without mucosal targeting, it is unlikely that immune cells will reach the cancers in vivo [13–15]. To overcome these issues, we proposed a new type of peptide mucosal vaccine formulated with an adjuvant that enhances cellular immune responses and specifically targets mucosal sites. In addition, we focused on the p53 protein as the antigenic target since the p53 protein is highly mutated in CRC, with *TP53* missense alleles present in about 60–65% of human CRCs. *TP53* missense mutations are important CRC targets as they contribute to tumor development and progression by impairing wild-type p53 function [16, 17]. Some p53 mutants appear to have gain-of-function effects, such as promoting cancer cell proliferation, invasion, and metastasis, thereby promoting cancer progression [16, 17].

We examined the use of a mutant p53 vaccine in a mouse CRC model based on combined mutations in the murine Apc, Kras, and *TP53* genes (i.e., APCΔ580, KRAS-G12D and Trp53-R270H triple mutants). The *TP53* abnormality is inducible, so we could assure the induction of p53 before we initiated immunization with the candidate vaccine. Also, the human R273H *TP53* missense abnormality is one of the most common abnormalities in human CRC [16, 17]. In this study, we use a vaccine approach with a peptide corresponding to the R270H sequence (the mouse ortholog to human R273H) and a mucosal adjuvant to drive epithelial immunity. We also included recombinant p53 protein (rp53) to attempt to overcome tolerance. This vaccine (p53/NE) was administered by intranasal application into mice three days after tamoxifen induction of mutant *Trp53* to examine its ability to induce immune responses as well as assessing tumor features and survival.

## Materials and methods

### Preparation of intranasal protein/peptide vaccine

An R270H (the mouse ortholog to human R273H) peptide was selected for testing and acquired from GenicBio Limited (Shanghai, China). The peptide sequence is as follows: SGNLLGRNSFEVHVCACPGRDRRTE. Oil-in-water mucosal adjuvant (MA), a formulation of nanoemulsion (NE), was prepared with high-speed shed homogenize as described [18]. The R270H peptides at 0.01 µM and 1.25 µg/ µL of wild-type p53 recombinant protein (Proteintech, Rosemont, IL) were combined in 20% NE as a novel intranasal p53/MA formulation.

### Determination of R270H peptide stability in the vaccine

The stability of the R270H peptide in MA was analyzed by UPLC. UPLC analysis was performed in a Waters Acquity UPLC system equipped with PDA spectrometer. PBS and 20% MA alone were used as controls.

### Mouse CRC model

**Fig. 1:**
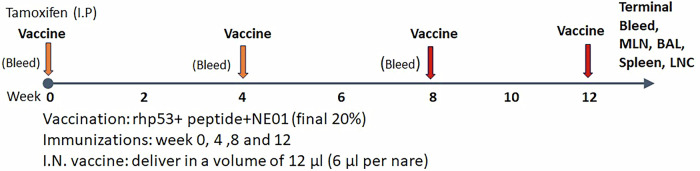
Mice were received four doses of the intranasal vaccine at four-week intervals. Schedule of intranasal administration of vaccines to mice.

All animal use protocols were approved by the University of Michigan Institutional Animal Care and Use Committee (IACUC). CDX2P-CreERT2Apcflox/+ KrasLSL-G12D/+ Trp53R270H/flox mice (previously referred to as AKP270/fl and abbreviated in this study as AKP mice) were previously described [19, 20]. This mouse CRC model is based on combined mutations in the Apc and *Trp53* tumor suppressor genes and the Kras oncogene, with Cre-mediated gene targeting via the CDX2P-CreERT2 transgene and tamoxifen (TAM) treatment to activate Cre function in colon epithelial cells [1]. AKP mice aged 1.5–4 months were injected intraperitoneally with TAM (Sigma-Aldrich, St. Louis, MO) dissolved in corn oil (Sigma-Aldrich). The mice received two daily doses of TAM, with each dose being 70 mg/kg. The AKP mice were immunized every 4 weeks with p53/NE vaccine or control vehicles (NE or PBS) after 3 days following TAM treatment (Fig. 1). The animals were randomly assigned to one of three groups: p53/NE vaccine, NE alone, and PBS control using a computer-generated random sequence and euthanized upon reaching humane endpoints defined as body weight dropping by 15%, blood stools, or lethargy [21].

### Vaccination of AKP transgenic mice to evaluate anti-tumor activity

The active immunization group received R270H peptides in NE adjuvant, administered in 15 µL into each nare (30 µL total) three days after TAM administration, then three times subsequently at 4-week intervals. Control groups received either NE or PBS. This scheme outlined in Fig. 1. The animal groups and treatments are detailed in Table 1.

**Table 1:** p53 vaccination groups and treatments.

Groups	rP53 +peptide	PBS	60%NE
NE 7 mice		8 µl	4 µl
rP53 + p53 R270H peptide + NE 9 mice	15 µg rP53 + 10 µg R270L in 8 µl		4 µl
PBS 9 mice		12 µl	

p53-R273-WT: SGNLLGRNSFEVRVCACPGRDRRTE = mouse WT.

p53-R273H: SGNLLGRNSFEVHVCACPGRDRRTE = mouse R270H.

### Analysis of p53/NE-specific immune responses

Cytokine (IFNγ, IL-2, IL-10 and IL-17) levels from p53/NE-specific splenocytes and lymph nodes were measured using Milliplex Map mouse cytokine/chemokine multiplex assay kits (Millipore, Burlington, MA). Briefly, after a 96-well plate was washed with wash buffer, samples, controls and diluted standards were added to the plate together with antibody-immobilized premixed beads, and incubated with shaking overnight at 4 °C. The plate was then washed, and the detection antibodies were added. After a 1-h incubation with shaking at room temperature, streptavidin-phycoerythrin was added to each well and the plate was incubated for 30 min at room temperature with shaking. The plate was then washed, 150 μL of Sheath fluid was added to all wells, and the cytokine levels were measured by Luminex 200 and then analyzed by Belysa (Millipore). Values are the means of at least two independent determinations (with three replicates within each experiment).

The levels of the cytokines were confirmed by ELISpot assay (MABTECH, Cincinnati, OH). Briefly, splenocytes and cervical lymph node cells were cultured with either 80 µg/ml R270H peptides or media (as a control) then added into the plate and immobilized with IFN-γ or IL-17 monoclonal capture antibodies. After 48 h of incubation, cytokine detection antibodies (R4-6A2-biotin and streptavidin-ALP) were added, respectively, and plates were visualized by staining reagents (BCIP/NBT). The plate was evaluated using an AID ELISpot reader system. Data are expressed as spot-forming cells per million cells.

### Histology

After mice were euthanized, the thoracic cavity was opened, and a 22-gage catheter was inserted into the trachea and fixed with a ligature. Colons with tumors were harvested for routine overnight fixation (in 10% formalin), processing, and staining with hematoxylin and eosin (H&E) for use in tumor quantification and morphologic analysis. All images were captured using a scanner, a Leica Aperio AT2® with the specifications of magnification 20× objective equivalents, 50,000 pixels/inch resolution, brightfield illumination. Tumor differentiation was determined by the percentage of glandular structures [22, 23]. Briefly, the glandular structures ≥50% were classified as high differentiation; the glandular structures between 49% and 5% as moderate differentiation; and the glandular structures <5% as low differentiation. To elimination of observer bias, the images were assessed by two independent laboratory members who are blind to mouse group assignment information.

### Immunohistochemistry of CD8

CD8 staining was performed on Formalin-Fixed Paraffin-Embedded sections of tissue. Tissue sections were deparaffinized in xylene and rehydrated in ethanol and water, following by using heat-induced epitope exposure; following quenching and blocking, the tissues were incubated with primary antibody Anti-CD8 alpha antibody (dilution 1:800, EPR21769, Abcam, Cambridge, UK) and then HRP-Goat anti-Rabbit Secondary Antibody (Part NO. HC005R in PK10017 kit).

### TUNEL assay

Following the manufacturer’s protocol, paraffin slides (5 μm thick) were examined with One-Step TUNEL Apoptosis Kit (C11026, RiboBio). Briefly, paraffin slides were rehydrated and permeabilized with dilute proteinase K. After quenched with 3% H_2_O_2_ to inactivate the endogenous peroxidases and equilibrated with TdT equilibration buffer, tissues were labeled with TdT enzyme, detected with Conjugate Buffer, and developed with DAB solution.

### Statistical analysis

Continuous data were presented as the mean ± SEM. Student’s *t* test with Welch’s correction, Mann–Whitney, one-way ANOVA and Log-rank (Mantel–Cox) were utilized to define statistical differences. *P* values less than 0.05 were considered significant. The sample size (number of mice per group) was calculated based on our earlier data and a program developed by Rollin Brant at the University of British Columbia. Therefore, the estimated sample size required per group is 7 (α = 0.02, power = 0.80).

## Results

### p53/NE vaccination induces antigen-specific cytokine responses in splenic and lymph node cells

**Fig. 2:**
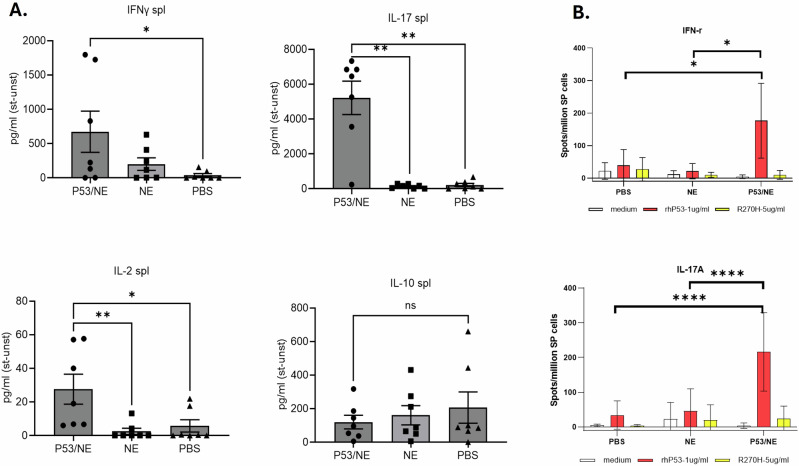
The cytokine production from splenic lymphocytes. Spleens were resected from the mice vaccinated with P53/NE, or the controls (PBS, NE). The production of cytokines from splenic Lymphocytes was determined by Luminex assay (**A**) and ELISpot assay (**B**). *p < 0.05; **p < 0.01: compared with the controls (PBS or NE).

The induction of neoantigen-specific cytokine responses is critical in anti-tumor vaccines, including tumor-inhibitory Th1 cytokines such as IFNγ, IL-2, and IL-10 [24, 25]. After administration of the p53/NE vaccine, luminex assays were used to measure the levels of IFNγ, IL-17, IL-10 and IL-2 in lymphocytes isolated from the spleen and lymph nodes of tumor-bearing animals. As compared with control mice immunized with saline or adjuvant alone, p53/NE vaccinated mice significantly increased the production of IFNγ, IL-17 and IL-2; however, the vaccine had no effect on IL-10 levels (Fig. 2A). The Luminex assay results were verified using an ELISpot assay, which also showed the p53/NE vaccine could enhance p53-specific IFNγ and IL-17 (Fig. 2B).

**Fig. 3:**
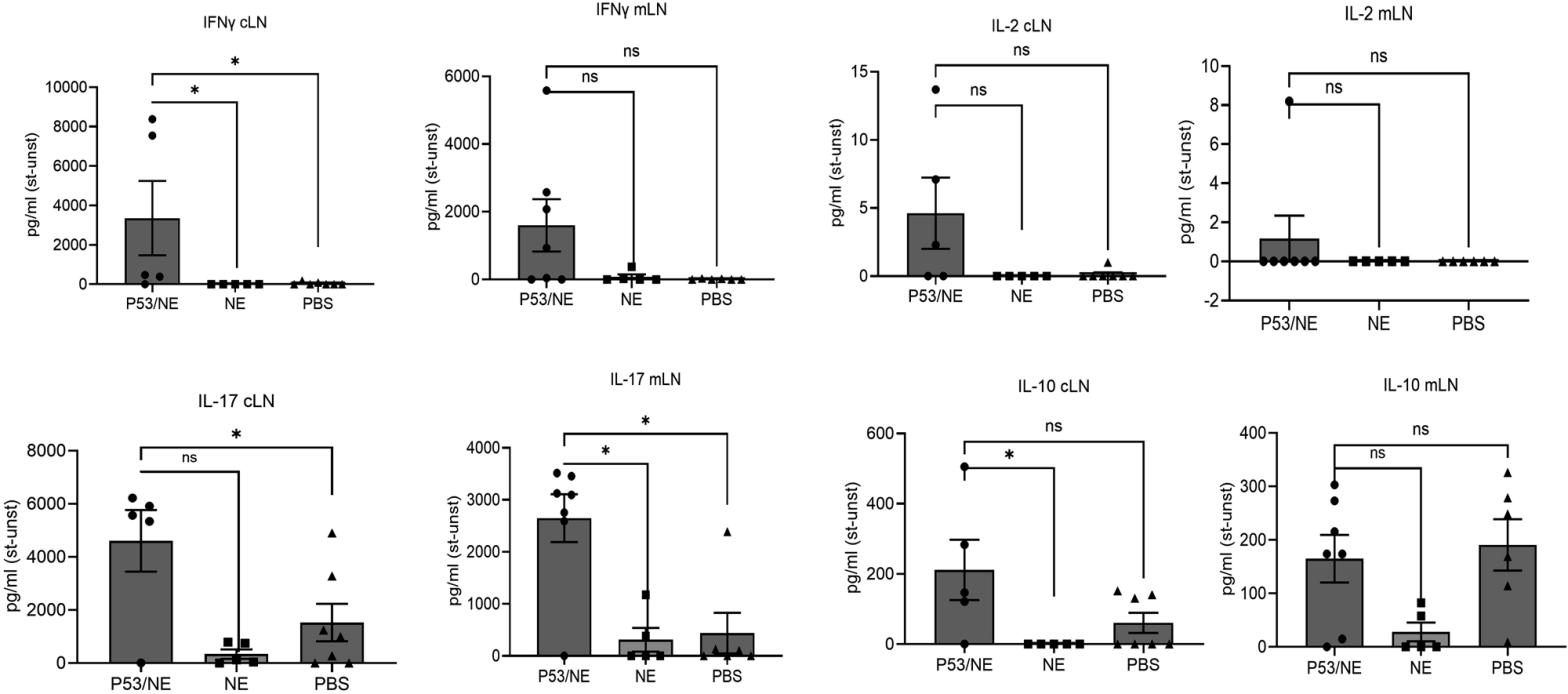
The cytokine levels in mesenteric lymph nodes (mLN) and cervical lymph nodes (cLN). mLN and cLN were obtained from the mice vaccinated with P53/NE, or the controls (PBS, NE). The production of cytokines (IFNγ, IL-17, IL-10 and IL-2) from the lymph nodes was determined by Luminex assay. *p < 0.05, compared with the controls (PBS or NE). ns not significant.

Cervical lymph nodes (cLN) are not a common site of metastases for CRC in patients. Based on the nasal immunization approach, we postulated cLN cells might initiate the *TP53* immune responses that then spread to other mucosal sites, such as mesenteric and colonic lymph nodes [26]. We therefore assessed selected cytokine responses to the p53/NE vaccine in cLN and mesenteric lymph nodes (mLN). The p53/NE vaccination significantly increased neoantigen-specific IFNγ and IL-17 responses in both mLN and cLN, as compared with control vaccinations (Fig. 3). The IL-2 response to the vaccine for both mLN and cLN was also higher in the p53/NE vaccine group than the controls (Fig. 3), but this failed to reach significance, possibly due to the small number of cells available for analysis. We also found the p53/NE vaccination promoted the production of cLN IL-10 as compared with NE or PBS, but it did not alter mLN lymphocyte IL-10 generation (Fig. 3).

### Impact of p53/NE vaccination on serum humoral immune responses

**Fig. 4:**
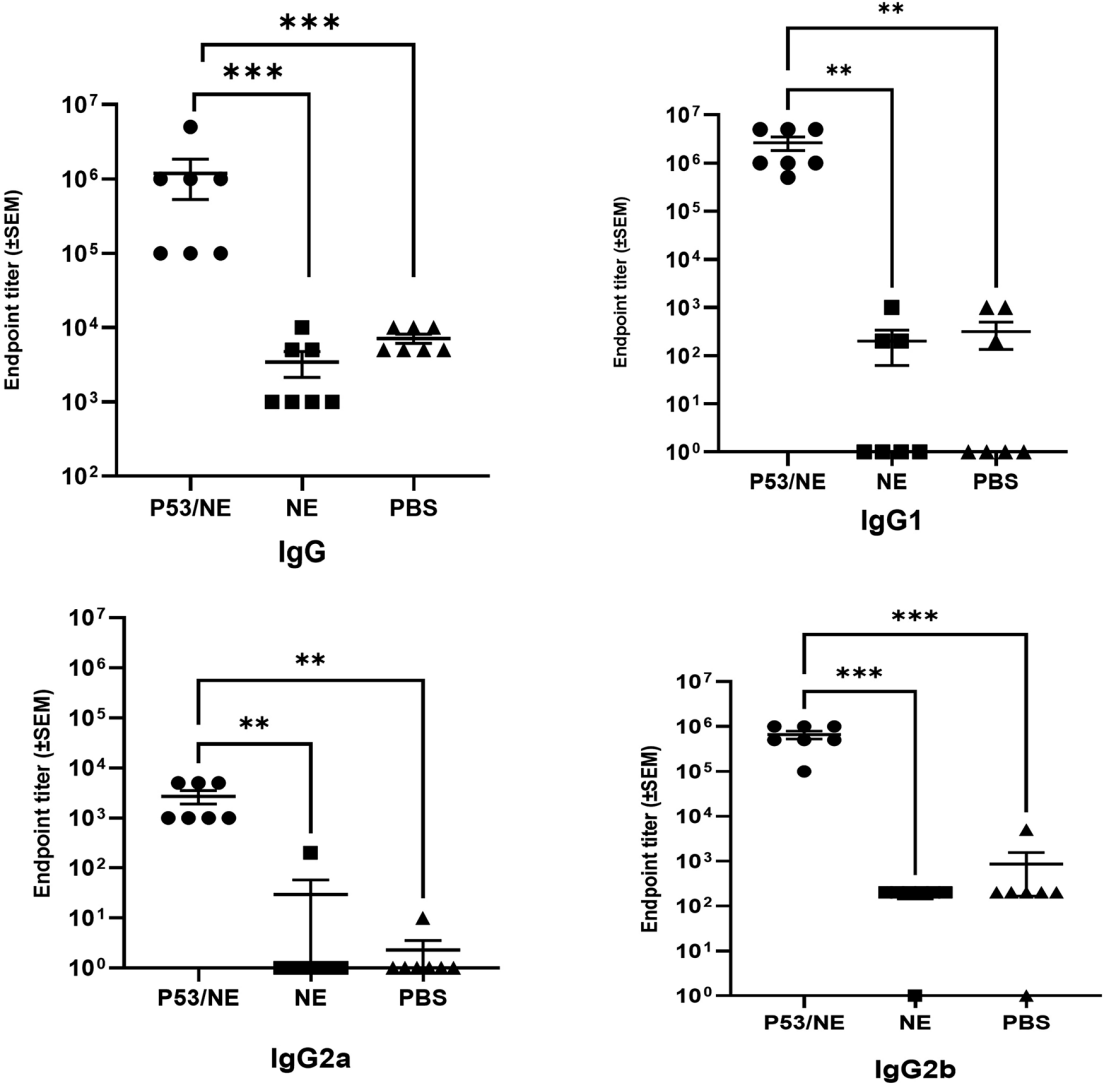
Enhancement of humoral responses by intranasal P53/NE vaccination. After mice were vaccinated by P53/NE, the antigen-specific (anti-rhP53) antibodies IgG(H + L), IgG1, IgG2a and IgG2b in serum were determined by endpoint assays. **p < 0.01; ***p < 0.001, compared with the controls (NE or PBS).

Cancer immunotherapy can kill tumor cells leading to the release of tumor antigens. This can stimulate B cells to produce antibodies, and although not thought to be a primary anti-tumor approach, the B cells can lead to targeting cancer cells, promoting their destruction by NK cells or complement enhancing anti-tumor immunity [27, 28]. To assess the induction of anti-Trp53 specific antibodies in these animals we used endpoint titer assays to measure serum p53-specific IgG(H + L), IgG1, IgG2a and IgG2b. Overall titers of IgG(H + L), IgG1, IgG2a and IgG2b antibodies to mutated peptide and whole p53 protein were significantly increased in p53/NE vaccination group when compared with the control animals (NE or PBS) (Fig. 4). This pattern of antibody isotype response suggested a Th1-type immune bias.

### p53/NE vaccination effect on tumor mononuclear cell infiltrates

**Fig. 5:**
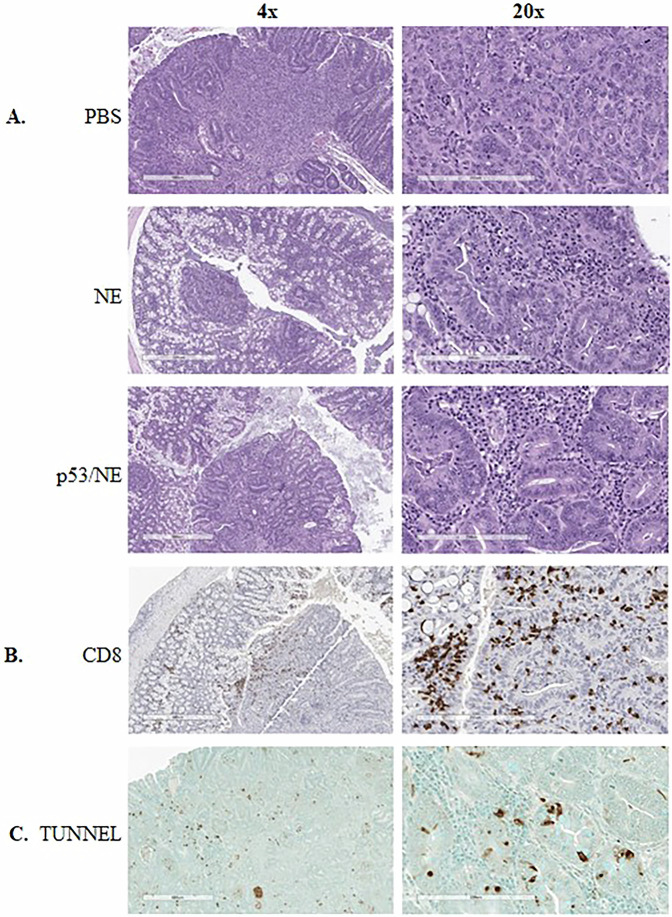
Mononuclear cell infiltration and immunohistochemical results in tumors. **A** Mice were inoculated with P53/NE, NE alone, or PBS and euthanized when the body weight dropped by 15%, then tumors were removed. Cross sections of damaged intestine were stained with hematoxylin-eosin (H&E), and infiltrating mononuclear cells were measured. **B** CD8 positive staining was detected by anti-CD8α antibody and a representative CD8 staining from p53/NE-treated tumor tissues was shown. CD8 positive cells were obviously increased in mice treated with p53/NE, compared with mice treated with NE or PBS (*p* < 0.0001). **C** Apoptotic cells were detected by the TUNNEL assay and a representative TUNNEL assay result from p53/NE-treated tumor tissues was shown. Apoptotic cells were increased in mice treated with p53/NE, compared with mice treated with NE or PBS, but the difference did not reach a significant point (*p* > 0.05).

Mononuclear immune cell infiltration in tumors is thought to be a key factor that enhances the beneficial outcome from cancer immunotherapy [29, 30]. Blinded examination of tumor sections from immunized animals showed marked increases in mononuclear cell infiltrates than in cancers from control animals (Fig. 5A). The quantitative analysis of cellular infiltration in the three groups are shown in Table 2. The percentage of mononuclear cells infiltrating the tumors is significantly increased in p53/NE immunized group compared to NE or PBS (*p* < 0.01). CD8^+^ T cells were the most abundant in the infiltration and the extent of CD8 infiltration is marked increased in p53/NE group vs. NE or PBS (*p* < 0.0001, Fig. 5B). The infiltrates were diffused throughout the tumor and spatially associated with apoptotic cancer cells (Fig. 5C). Despite this, TUNNEL assay showed only 8-10% apoptotic cells in all tumors without significant difference among active and control groups (*p* > 0.05).

**Table 2:** Summary of mononuclear cell infiltration.

Groups	None	25% or less	50% to 75%	100%
		Infiltration	Infiltration	Infiltration
NE	4/7	3/7	0/7	0/7
PBS	4/7	2/7	1/7	0/7
p53/NE	0/7	2/7	3/7	2/7

### p53/NE vaccination impact on tumor size and animal survival

**Fig. 6:**
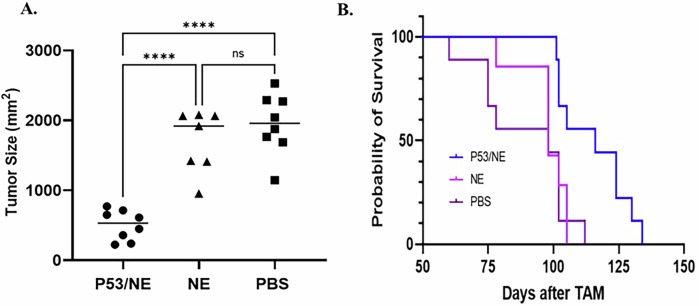
Outcome of p53 vaccinated animals. **A** Tumor size. The tumor size was calculated at the end of experiments when the mouse body weight dropped by 15%. ****p < 0.001. ns not significant. **B** Comparison of the survival rate of animals in each group, p53/NE vs. NE or PBS *p < 0.05; NE vs. PBS *p* > 0.05.

Tumor size and animal survival are important measures of the efficacy of CRC treatment (1.2). Our data revealed the p53/NE vaccination significantly reduced the size of tumors compared with NE and PBS controls (*p* < 0.0001, Fig. 6A), and the number of tumor nodules and metastases was less. In addition, mice receiving p53/NE vaccine had significantly longer survival than NE alone or PBS control) (*p* < 0.05, Fig. 6B). Survival was dependent on humane endpoints assessment, so this was not surprising. Furthermore, tumor cells from animals receiving p53/NE vaccination appeared better differentiated histologically than those from control animals; however, this difference did not reach significance (*p* > 0.05, Table 3).

**Table 3:** Tumor cell differentiation in p53 vaccinated animals and the controls.

	Location(s)		Tumor volume (mm³)	Neoplasm differentiation		
	Caecum	Proximal colon	(Average)	Low	Medium	High
p53/NE	4/8	5/8	477	0/8	3/8	5/8
NE	7/7	7/7	851	0/7	4/7	3/7
PBS	8/8	8/8	975	0/8	6/8	2/8

## Discussion

These studies target mutated p53 as a neoantigen in CRC. While the p53 protein is mutated in two-thirds of CRCs, it previously has not been shown to be an effective target for immunotherapy. To overcome this problem, we took a different approach to induce anti-p53 immunity. This involved developing a vaccine based on p53 peptides with an amino acid change associated with human colon cancer (R273H). Importantly, we also included wild-type p53 protein to include T cell epitopes not included in the mutated peptide sequences, The vaccine was administered with a mucosal adjuvant, NE, to enhance immunogenicity and to break tolerance to p53 and target immunity to mucosal surfaces. We evaluated this novel p53/NE vaccine in a conditional mouse CRC model based on ApcΔ580, KrasG12D and Trp53-R270H triple mutant. Our results in this initial study show that this p53/NE vaccine induced cellular immunity to p53, with an enhanced inflammatory state in the tumors. The immune and inflammation effects observed linked to the inhibition of tumor growth in the mice and while prolonged animal survival.

Prior to our studies and findings, induction of immunity to p53 in CRC has not appeared to be a promising approach. While two-thirds of CRCs express missense mutant p53 proteins [16, 17], p53 vaccines have not been assessed extensively in human cancers. The most recent human study of a p53 vaccine was reported in a Phase 2 clinical trial 5 years ago in small cell lung cancer and involved TP53-transfected dendritic cells as a vaccine [31]. The vaccine was administered to 69 patients with recurrent small-cell lung cancer to enhance the efficacy of paclitaxel, but despite inducing immune responses in 20% of subjects, it did not improve the overall response rate [31]. Vaccines using long peptides (LPs, 28-35 aa) corresponding to driver gene mutations in *TP53* and KRAS have also been tested in a mouse model of CRC [32]. LP vaccination was able to induce polyvalent, multifunctional, and mutation-specific effector T cells capable of targeting tumors. Paradoxically, the vaccine accelerated tumor growth, and this appeared to be related to the upregulation of regulatory T cells [32]. The p53 LP vaccines were also studied in murine models of CRC by two other groups [33, 34]. While p53-specific immune responses were demonstrated, no reduction in tumor size or prevention of tumor occurrence were shown [33, 34]. Finally, a Phase I/II clinical study using a recombinant canary poxvirus vaccine encoding wild-type human p53 in 15 patients with advanced colorectal cancer [35] showed p53-specific immune responses in only two patients, and no antitumor activity [35]. Thus, the prior record of p53-based cancer vaccines has not been promising.

The anti-tumor effects of the vaccine used here may be attributable to several p53/NE-related immune responses. Firstly, p53/NE vaccine promoted the levels of IFNγ and IL-2 in response to the mutated peptide but did not enhance IL-10 production. This could have biased the T cell immune response in favor of a Th1profile, enhancing tumor cytotoxicity to mediate cancer regression [36]. This phenomenon has been previously demonstrated in CRC [37, 38]. p53/NE also induced anti-p53 specific antibodies including mouse subclasses IgG1, IgG2a and IgG2b, which can further bias Th1-type immunity [39, 40]. This suggests Th1-biased T cell responses could be important in the effect of this vaccine. The p53/NE vaccine also appeared to stimulate production of IL-17, and while the effects of increasing IL-17 in tumor immunity are controversial, the upregulation of IL-17 can enhance anti-tumor immunity by recruiting and activating effector immune cell subsets including dendritic cells, neutrophils, CD8^+^ T cells, and NK cells [41]. Furthermore, IL-17 can promote IFNγ-producing cytotoxic type 1 subset or Th1-type responses [41, 42]. We have observed that the p53/NE enhanced mononuclear cells infiltration, specifically increased numbers of CD8^+^ T cells in tumors, which may be either pro- or anti-tumor in its effect [43, 44]. Though the importance of this finding remains unclear, the presence of T-cell tumor infiltrates is associated with innate antitumor immunity and may benefit using therapeutics that block immune checkpoints.

The density of antigen-specific effector T cells within the tumor microenvironment and invasive margin is a predictor of survival in patients with CRC, and the idea that pre-treatment adaptive immune responses and immune infiltration directly influence the natural history of different malignancies is consistent across studies [45, 46]. Furthermore, increased CD8^+^ T cell infiltration in the tumor microenvironment was directly associated with the benefit of PD-1 blockade [47, 48]. Together, these data support that p53/NE vaccine combined with PD-1 blocking therapy can potentially enhance the efficacy. It has been noted that cancer patients who respond well to immunotherapies or ICI usually have an abundance of tumor tissue-infiltrating immune cells [49]. In CRC, tumor-infiltrating immune cells, such as tumor-associated macrophages, affect the prognosis and efficacy of chemotherapy and immunotherapy and impact tumor angiogenesis, progression, and metastasis [50–52]. The mononuclear cells infiltrate in our animal models were associated with reduced tumor burden, so this result is in line with the immune cell infiltration of CRC being associated with a more favorable prognosis and less metastatic invasion [46]. Thus, it appears that the p53/NE vaccine is able to generate multiple anti-CRC immune activities, which has not been previously reported in p53 vaccines.

An interesting feature of our vaccine is the incorporation of a NE with the p53 peptide antigens. The NE we employed has been demonstrated as well tolerated in human trials and can induce Th1, Th17, and IFNγ immune responses, producing an immune phenotype associated with the development of effective anti-tumor responses [18, 24, 29, 30, 41, 42]. Furthermore, NE can stabilize the vaccine and facilitate its uptake via the mucosal cells [18, 29]. Mucosal vaccination is considered promising for tumor immunity because it can induce a persistent mucosal immune response that would provide surveillance for future transformed cells.

Several aspects of our vaccine are not currently optimized. The length of the p53 peptide used in our vaccine is 25aa. This is a bit shorter than the LP p53 vaccines tested in previous studies [32–34]. Though we are not sure if a short peptide is superior to a longer one, it may facilitate T cell MHC presentation without requiring processing and may be a significant factor in the efficacy of peptide vaccines. Also, we included wild-type p53 protein in our vaccine, with the intent to help break tolerance to p53. Whether this is beneficial remains to be seen, and the wild-type p53 protein might raise concerns about autoimmune activity. On the other hand, breaking tolerance to p53 protein could provide an important first step in inducing anti-tumor immunity.

In conclusion, an experimental p53/NE vaccine was effective in inducing anti-p53 immunity. This approach also demonstrated an ability to suppress CRC growth and improve survival in an inducible mouse CRC model with APCΔ580, KRAS-G12D and p53-R270H triple protein mutants. The anti-tumor effect is associated with enhanced antigen-specific cellular and humoral immune responses as well as CD8^+^ T cell tumor infiltrates in the animals receiving p53/NE vaccine. This data suggests further investigations are warranted to understand the role of p53 immunity in controlling CRC.

## Data Availability

Data openly available in a public repository that issues datasets with DOIs.
